# Chemical Characterization and Biological Activity of the Mastic Gum Essential Oils of *Pistacia lentiscus* var. *chia* from Turkey †

**DOI:** 10.3390/molecules25092136

**Published:** 2020-05-02

**Authors:** Nurhayat Tabanca, Ayse Nalbantsoy, Paul E. Kendra, Fatih Demirci, Betul Demirci

**Affiliations:** 1 United States Department of Agriculture, Agricultural Research Service, Subtropical Horticulture Research Station (SHRS), Miami, FL 33158, USA; Paul.Kendra@usda.gov; 2 Department of Bioengineering, Faculty of Engineering, Ege University, Bornova, Izmir 35100, Turkey; 3 Department of Pharmacognosy, Faculty of Pharmacy, Anadolu University, Eskisehir 26470, Turkey; demircif@gmail.com (F.D.); betuldemirci@gmail.com (B.D.); 4 Faculty of Pharmacy, Eastern Mediterranean University, Famagusta 99628, Cyprus

**Keywords:** Anacardiaceae, terpenoids, α-pinene, myrcene, β-pinene, GC-MS, chiral-GC, bioactivity, cytotoxicity, antimicrobial, Mediterranean fruit fly

## Abstract

The essential oils (EOs) were isolated by hydrodistillation from wild and cultivated *Pistacia lentiscus* L. var. *chia—*mastic gum tree (Anacardiaceae) from two natural habitats, namely from Cesme–Uzunkoy (1) and Mordogan (2), and one cultivated source, Cesme–Germiyan (3), in Izmir, Turkey. This comparative study evaluated the chemical composition and biological activity of mastic gum essential oils (MGEOs). For this purpose, MGEOs 1–3 were analyzed by gas chromatography–flame ionization detection (GC-FID), gas chromatography–mass spectrometry (GC-MS), and chiral GC for α-pinene. Laboratory assays were conducted to assess for potential in vitro cytotoxicity (multiple in vitro cancer cell lines), antimicrobial properties (five bacterial species and yeast), anti-inflammatory activity (inhibition of inducible nitric oxide synthase, iNOS), and the attraction of *Ceratitis capitata* (Mediterranean fruit fly, medfly), respectively. Chemical analysis indicated that MGEOs 1 and 2 were rich in α-pinene (56.2% and 51.9%), myrcene (20.1% and 18.6%), and β-pinene (2.7% and 3.1%), respectively; whereas MGEO-3 was characterized by a high level of α-pinene (70.8%), followed by β-pinene (5.7%) and myrcene (2.5%). Chiral GC analyses showed that concentration ratios between (−)/(+)-α-pinene and (−)-α-pinene/myrcene allowed for differentiation between wild and cultivated MGEO sources. In biological assays, MGEOs 1–3 did not exhibit significant antimicrobial effects against the pathogens evaluated and were not strong attractants of male medflies; however, all three MGEOs displayed a dose-dependent inhibition of iNOS, and MGEOs 1 and 2 exhibited selective in vitro cytotoxicity against human cancer cells. These results suggest that wild-type mastic gum oils from Cesme and Mordogan (MGEOs 1 and 2) are potential sources of beneficial products and warrant further investigation.

## 1. Introduction

Mastic gum is a resin obtained as a trunk exudate of the mastic tree *Pistacia lentiscus* L. var. *chia* (Anacardiaceae). The mastic tree is native throughout the Aegean and Mediterranean regions and is cultivated extensively in southern Chios, a Greek island in the Aegean [1,2,3,4,5,6,7,8]. Since ancient times, mastic gum has been utilized for the treatment of digestive and gastric diseases as well as *Helicobacter pylori*, the principal cause of peptic ulcer [2,3,4,5,6,7,9,10]. Mastic gum is a complex mixture of different phytochemical groups, including mono-, sesqui-, and triterpenoids, as well as phenolic compounds, many of which are strongly linked to biological activities, displaying antimicrobial, antioxidant, anti-inflammatory and anticancer properties [4,5,6,7,9,10,11,12,13]. Recent studies demonstrated that the *P. lentiscus* resin dichloromethane extract inhibited acetylcholinesterases [6]. Mastic gum was proposed for cardiovascular protection due to its preventive effect of low-density lipoprotein (LDL) oxidation [7,14]. Chewing mastic gum exhibited significant antibacterial activity against *Streptococcus mutans*, resulting in good oral health by preventing dental caries [15,16]. Mastic gum has a remarkable reputation in food and pharmaceutical products due to its pleasant aromatic and therapeutic properties. For example, it is incorporated into baked goods (cakes, cookies, tsoreki) and milky desserts/sweets, including Turkish delight, halva, ice-cream, yogurts, candies, and chewing gum [4,9,11,17], and also in alcohol-containing drinks (mastiha liquor, ouzo), beverages such as soumatha, and some specialty mastic-flavored coffees. Additionally, mastic gum products are used in cosmetic formulations such as soap, body lotions, shampoos, toothpastes, mouthwashes, and surgical adhesive strips [11,17,18,19,20].

Due to its economic value, Chios mastic gum is considered a Product of Protected Designation of Origin (PDO), subject to No. 123/1997 (L0224/24-1-97) of the European Union, Traditional Specialty Guaranteed (TSG), and Protected Geographical Indication (PGI). As such, mastic gum is described as an agricultural and food product, allowing growers to promote their products in the market and assure consumers of the quality of mastic products [5,7,9,11,18,21] and the traditional practice of mastic gum collection in Chios has been listed on the Representative List of the Intangible Cultural Heritage of Humanity (ICH) since 2014 [7]. In 2015, mastic gum was designated by the European Medicines Agency (EMA) as a natural medicinal product for the treatment of mild dyspeptic disorders, and minor skin wounds [5,7,9,18,21]. It is worth mentioning that mastic gum is documented in the European Pharmacopoeia (Monograph 1876), which reports requirements for its physical properties, and describes its thin-layer-chromatography-based identification [5,7,22].

**Figure 1:**
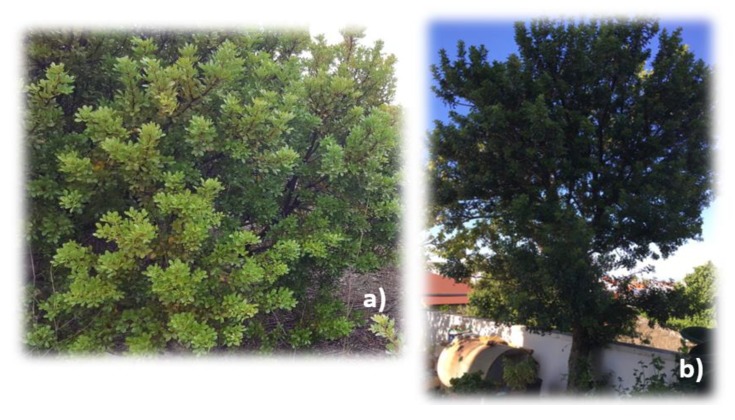
Wild mastic trees in: (**a**) Ovacik Village, Cesme Penunsula; and (**b**) Mordogan, Karaburun Penunsula. Photos courtesy of Mustafa Ozer (**a**) and N.T. (**b**).

The mastic tree grows naturally in Izmir on the Cesme–Urla–Karaburun Peninsula in the Aegean region of Turkey (Figure 1), which faces the Greek island of Chios to its west [23,24]. Commonly, the mastic tree is known as “sakiz agaci” and mastic gum as “damla sakizi” in Turkey [23,25].

**Figure 2:**
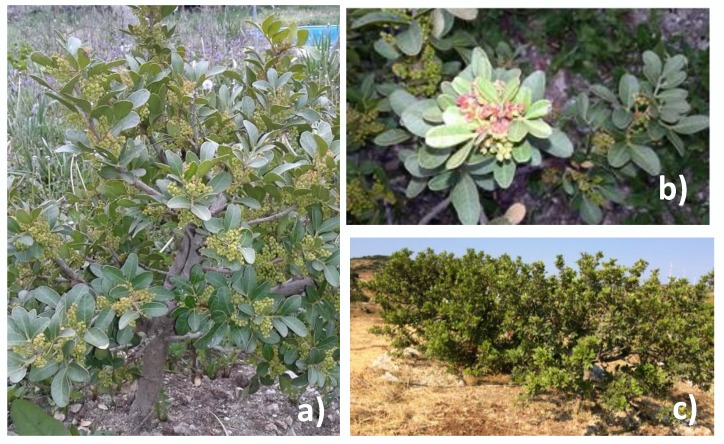
Cultivated mastic trees in Mordogan, Karaburun Peninsula, (**a** and **b**) and Nezih Ozture Farm, Cesme Peninsula (**c**). Photos courtesy of Mustafa Ozer (**a** and **b**) and A.N. (**c**).

The Turkish Foundation for Combating Soil Erosion (TEMA), non-governmental organization for reforestation and the protection of natural habitats in Turkey, has been leading new projects to protect the mastic trees in their natural habitats, and to establish new plantations in the Cesme–Urla–Karaburun Peninsula to revive gum production [26,27] (Figure 2). 

Although the Cesme Peninsula is known to be home to mastic [28], the trees also grow indigenously in Mordogan, a coastal town of Karaburun Peninsula where the landscape is formed from the north of the Urla Peninsula, and mastic tree is locally known as “sakizlik” in Mordogan. Leaves with small branches are used to prepare a decoction for skin infections, gastric ulcer, and abdominal pain by the local communities [29]. For example, mastic gum mixed with tahini-halva was suggested for patients who suffer from severe peptic ulcers. Due to its pleasant aroma and antimicrobial properties, mastic gum or tree leaves are commonly added to jams, jellies, and pickled products for extending the food longevity by local farmers and residents in Karaburun [29]. However, despite the cultural importance and natural occurrence of the mastic tree in the Karaburun Peninsula, no studies on the volatile composition of mastic gum collected from sites like Mordogan were reported, to the best of our knowledge. Therefore, a comprehensive study was initiated for the first time, to investigate the chemical composition of mastic gum essential oils (MGEOs 1–3) collected from two natural habitats (Cesme-Uzunkoy (MGEO-1) and Mordogan (MGEO-2)), to compare the composition of MGEOs obtained from these wild trees to MGEO from cultivated tree (Cesme-Germiyan (MGEO-3)), and to evaluate the biological activity of MGEOs 1-3 from three sources. The latter included laboratory assays to assess for cytotoxic, antimicrobial, and anti-inflammatory properties by the inhibition of inducible nitric oxide synthase (iNOS) properties, as well as for the attraction of a major insect pest, the Mediterranean fruit fly, or medfly, *Ceratitis capitata* (Wiedemann) (Diptera: Tephritidae). 

## 2. Results and Discussion

### 2.1. Chemistry of Mastic Gum Essential Oils 

In this present study, mastic gums collected from two wild trees around 100-years old (MGEO-1 and 2) and one cultivated tree about 7-years old (MGEO-3) were hydrodistilled, and, subsequently, oils were analyzed by gas chromatography-flame ionization detection (GC-FID) and gas chromatography-mass spectrometry (GC-MS). The pale-yellow oils were obtained with the yields of 2.17%, 2.0%, and 2.25% (*v/w*) from MGEOs 1–3, respectively. Forty-eight to 57 compounds were identified, representing 99% to 99.2% of the total oils. The results show that oils obtained from the mastic gums of the wild-type trees were dominated by α-pinene (56.2% and 52%) and myrcene (20 and 19%), whereas the oil obtained from mastic gum of cultivated trees was rich in α-pinene (71%) and lower amounts of β-pinene (6%) and myrcene (3%). However, samples varied in terms of composition by chemical classes, including monoterpenes (77%–85%), oxygenated monoterpenes (6%), sesquiterpene hydrocarbons (5%–8%), oxygenated sesquiterpenes (1%–5%), diterpenes (1%–2%), and non-terpenoids (1%), as in Table 1. 

Table 2 summarizes the main components identified in *P. lentiscus* var. *chia* essential oils obtained from gums based on data from Scopus, Google Scholar, Pubmed, Web of Science and Chemical Abstracts from 1990 to 2020 using the keywords “mastic gum essential oil” and “*Pistatica lentiscus* var. *chia* essential oil” [5,33,34,35,36,37,38,39,40,41,42]. Mastic essential oils obtained from aerial parts such as leaves, branches, and fruits were excluded from the literature search process. As seen in Table 2, MGEOs were mainly characterized by α-pinene, myrcene, and β-pinene but also contained minor constituents such as limonene, linalool, verbenone, perillene, pinocarvone, *trans*-pinocarveol, etc. The essential oils of two wild-growing *P. lentiscus* gums (MGEOs 1 and 2) were qualitatively and quantitatively very similar, however, one cultivated oil (MGEO-3) differed regarding quantitatively on α-pinene, myrcene and β-pinene (Table 1). The differences could be related to wild versus cultivated mastic gum samples. Tree age could be another factor affecting the chemical composition of mastic gum essential oils. As a matter of fact, while our samples were only gums of the *P. lentiscus*, Kivcak et al. [23] studied to compare the composition of mastic oils obtained from twigs and leaves and authors also found significant differences between wild and cultivated oils. Other than the age of plant and wild versus cultivated trees, the environmental conditions and physicochemical properties of soil should be considered as well. In another study, mastic gum collected from the southwestern part of Turkey (Fethiye, Mugla) was characterized by β-pinene (38.7%), α-pinene (21.7%), pinocarvone (5.3%), α-ylangene (4.0%), limonene (3.8%), and *n*-nonanol (3.5%) [37]. These results were quite different than our results. Regarding the environmental conditions, Dogan et al. [43] investigated the soil–plant interactions of *P. lentiscus* trees in the western part of Turkey. The results showed that *P. lentiscus* grown on sandy-clay loam in Cesme and Mordogan (Izmir) was different than clay-loam soil in Fethiye (Mugla) region [43]. In this case, soil type and climate zones such as humidity, temperature, and light, might be affecting the chemical profile of MGEOs. Other than environmental conditions, the composition of mastic gum is influenced by extraction methods (Table 2) and the storage duration of mastic gums [36,40,44]. 

A comprehensive study of 45 authentic Chios MGEOs suggested that enantiomeric ratios of (−)/(+)-α-pinene ranging from 0.55:100 to 1:100 and (−)-α-pinene/myrcene ratios from 1.9:100 to 11:100 could be used to determine the quality of MGEOs and to identify adulteration of MGEOs with commercial mastic oils [40]. Based on this approach, we compared our results with the data presented in the literature [40]. The enantiomeric ratios are shown in Table 3. The rations of (−)/(+)-α-pinene were 0.58:100 to 0.60:100 and (−)-α-pinene/myrcene were 2.7:100 to 24:100 for MGEOs-1-3 (Table 2). In the present study, MGEOs 1 and 2 were in good agreement with those previously reported [40]. However, (−)-α-pinene/myrcene ratio in MGEO-3 was slightly higher than the authenticated MGEOs [40] due to a low percentage of the myrcene (2.5%) in sample MGEO-3. Furthermore, authors studied (−)/(+)-α-pinene and (−)-α-pinene/myrcene distribution in pharmaceutical and cosmetic products containing MGEOs and they found that the ratios ranged from 0 to 67:100 for (−)/(+)-α-pinene and 0 to 74:100 for (−)-α-pinene/myrcene [40], indicating that these products were adulterated with either racemic α-pinene or (−)-α-pinene or (+)-α-pinene predominated commercial chemical. It appears that (−)/(+)-α-pinene and (−)α-pinene/myrcene ratios differ between genuine and adulterated MGEOS, and pharmaceutical products containing mastic oil. Previous studies showed that α-pinene and myrcene content are dependent on the storage time [40]. For example, the level of α-pinene was raised to 72% in the mastic gum distilled 4–5 months after harvest, then increased to 79% in the gums distilled after 8–9 months. During storage duration, the proportion of α-pinene increased, and myrcene concentration decreased up to 11% for the gums distilled after 8–9 months. The relative percent of α-pinene and myrcene can be judged as an important contributor to authenticate the quality of MGEOs.

Rigling et al. [41] investigated the combination of olfactory detection and enantiomeric ratios of key odorant components in mastic oils. The authors found that the major volatile (+)-α-pinene and minor components myrcene, (−)-limonene, (+)-linalool, and perillene were the most characteristic contributors to mastic. It seems that the olfactory properties and chiral analysis can be useful tools for the authentication of mastic gum quality for consumers. Although we have not performed olfactory analysis, α-pinene, myrcene, limonene, linalool, and perillene were found in our samples MGEOs 1–3, which were considered as potential impact odorants for mastic gum. It should be noted that integrated multiple analytical methods exposing the fraudulent synthetic chemicals to MGEOs and mastic gum products are often needed. 

### 2.2. Biological Activities of Mastic Gum Essential Oils

The in vitro cytotoxic effects of MGEOs 1–3 were determined by an MTT (3-(4,5-dimethyl-2-thiazolyl)-2,5-diphenyl-2*H*-tetrazolium bromide)) assay using a broad range of human cancer cells: HepG2 (liver), SK-MEL-30 (melanoma), A549 (lung),253J-BV (bladder), CaCo-2 (colon), HeLa (cervix), PANC-1 (pancreas), PC3 (prostate), U87MG (glioblastoma), MCF-7 (breast), MDA-MB-231 (breast), non-tumorogenic cells HEK293 (embryonic kidney cells) and mouse macrophage cells RAW264.7. The cultured cells were treated with three different concentrations of MGEOs 1–3 (50, 5, 0.5 µg/mL) followed by incubation for 48 h at 37 °C (Supplementary Material Figure S1). Doxorubicin was used as a positive control. The results indicate that all samples were found to be active using the MTT method against all tested cancer cells but differed in IC_50_ (half maximal inhibitory concentration) values (Table 4). The microscopic analysis also showed that the morphological changes were comparable to the untreated control group (Supplementary Material Figures S2–S6). MGEOs 1 and 2 demonstrated the highest cytotoxicity against A549, RAW264.7 and SK-MEL-30 with the range of IC_50_ values from 5.25–9.20 µg/mL, whereas MGEO-3 showed the highest cytotoxicity against RAW264.7 and HeLa cells with IC_50_ values of 5.84 ± 0.68 and 7.621 ± 1.91 µg/mL, respectively. Interestingly, the MGEO 1 and -2 cytotoxicities against PANC-1, U87MG and MDA-MB-231 were higher than doxorubicin, whereas only MGEO 3 was against the cell line MDA-MB-231, respectively. According to the results observed, it is worthwhile to elaborate the cytotoxic effect of the essential oils/fractions or compounds in more detail, especially for the mode of action. The differences in cytotoxicity between MGEOs-1,2 and MGEO-3 could be related to their chemical profiles. The results suggest that myrcene may affect the overall activity in mixtures with other compounds synergistically, rather than in pure form. Furthermore, 

Macrophages play an important role in inflammatory diseases and can be stimulated by lipopolysaccharide (LPS) to produce pro-inflammatory cytokines and inflammatory mediators, such as prostaglandin E2 (PGE2) and nitric oxide (NO). NO is produced by inducible nitric oxide synthase (iNOS) and mediates many physiological processes in addition to inflammation [45]. In iNOS assays, MGEOs 1 to 3 were all effective for inhibiting the LPS-stimulated production of NO, with inhibition following a dose-dependent response (Table 5). IC_50_ values were found to be 11.6 ± 0.9, 13.75 ± 0.4 and 13.85 ± 0.5 μg/mL, for MGEOs 1–3, respectively (Table 6). The inhibition of these pro-inflammatory compounds is strategically important for the defense against inflammatory tissue damage and disease. Mastic gum consumption may also contribute to natural immunity and confer protection against inflammatory diseases [4].

The present in vitro cytotoxicity data were in accordance and supported the results reported with mastic oil samples from Chios island [4,12,46,47,48,49,50,51,52]. According to the literature research [7,36] and our results, terpenes present in the oil, such as α-pinene, myrcene, and β-pinene, constituting about 80% of the total, could attribute to the selective cytotoxicity. Spyridopoulou et al. [48] demonstrated that whole MGEO exhibited a stronger antiproliferative effect on human and murine colon cancer cells than any of its major constituents, including α-pinene, myrcene, β-pinene, limonene and linalool; however, α-pinene inhibited cell growth to a lesser extent [48]. In another study, [49] MGEO was reported to have a cytotoxic effect in vivo on lung cancer cells and results appear to provide a positive use in cancer prevention. MGEOs 1–3 also showed an inhibition effect on non-tumorogenic cells HEK293 which is accepted as normal cells since their genome has not undergone major rearrangements. Although the cytotoxicity of MGEOs on normal cell lines appears to be a disadvantage, their use in medical approaches is not a major barrier. Many of the current treatment strategies in the clinic have such an undesirable cytotoxic effect on healthy cells. Doxorubicin, a chemotherapeutic agent used in the clinic and used as a positive control drug in the study, has a cytotoxic effect on healthy cells, as seen in the results, but it is one of the widely used therapeutics in the treatment of cancer patients [50]. Accordingly, Spyridopoulou et al. [48] stated that mastic oil did not cause any toxicity in short-term oral administration in mice and no toxic effect was observed in another study examining the effect of mastic oil in different tissues. Attoub et al. [51] showed that mastic oil does not significantly change the redox or detoxification mechanisms in different tissues. Remarkably, the mastic oil or mastic water extract containing myrcene or α-pinene has been shown to have no genotoxic or mutagenic activities for in vitro and in vivo systems [48,51]. Therefore, mastic oil has always been suggested to have great potential in dietary nutrition use. In a comprehensive study, the anti-inflammatory activity was evaluated where mastic oil inhibited the production of pro-inflammatory agents by the inhibition of NO in the LPS-stimulated mouse macrophages cell line RAW264.7 [45], which was also confirmed in this present study. Similarly, Magkouta et al. [49] verified the anti-inflammatory activity of mastic oil through the suppression of NF-κB, indicating the potential anti-inflammatory effect of our samples. Another study showed that the use of mastic supplementation on patients with Crohn’s disease resulted in a significant reduction in the interleukin-6 (IL-6) level, which suggests that mastic gum may have potential use for patients with Crohn’s disease [53]. A recent study showed that microencapsulation with mastic gum can be used for sustained drug release [54].

The antimicrobial activity of MGEOs was examined against six microorganisms, including three Gram-negative (*Escherichia coli*, *Salmonella typhimurium*, and *Klebsiella pneumoniae*), two Gram-positive bacteria (*Staphylococcus aureus*, and *Listeria monocytogenes*), and the yeast *Candida albicans***,** respectively. As listed in Table 7, using the microdilution susceptibility assay, MGEO-1 showed weak inhibitory effects against all the tested human and food-borne bacterial pathogens tested except for *E. coli*. MGEO 2 and 3 demonstrated selective antibacterial activity against *K. pneumoniae* and *S. typhimurium*, respectively. As an antifungal agent, MGEO-1 was twice as effective against *C. albicans* than MGEOs 2 and 3. Compared to the standard controls such as chloramphenicol, amoxicillin, amphotericin B, the tested MGEOs were in general less active. The present antimicrobial results are comparable to previously reported studies [3,4,15,16,35,37,38].

The antimicrobial activity of the mastic oil was previously evaluated against *E. coli*, *Staphylococcus aureus* and *Bacillus subtilis* using the disk diffusion method [38]. Although the oil showed clear inhibition zones, the major compound, α-pinene, demonstrated resistance to *E. coli* and *B. subtilis*, and the second-highest compound, myrcene, did not inhibit *E. coli*, but it showed an intermediate response to *S. aureus* and sensitivity to *B. subtilis*. However, some of the other components, such as linalool, α-terpineol, and verbenone, showed relatively strong inhibitory activity on the growth of *E. coli*, *S. aureus* and *B. subtilis*, which is comparable to the oil itself [48]. The antimicrobial activity of MGEO could be attributed to the combination of several components rather than the main components or particular compounds, and the interactions of minor compounds should not be ignored. Studies showed that mastic gum has been linked to oral health due to its antimicrobial effect by reducing bacterial growth of *Streptococcus mutans* [15,16,55]. Recently, Chios mastic gum was used for the encapsulation of lactic acid bacteria *Lactobacillus casei*, which resulted in the extended shelf-life of fermented milk products [56].

**Figure 3:**
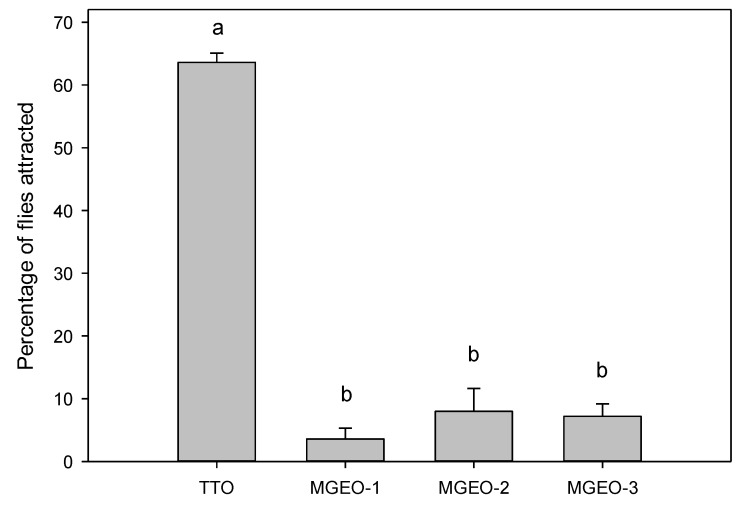
Attraction (mean ± SE) of male Mediterranean fruit flies to mastic gum essential oils (MGEOs 1–3) and tea tree essential oil (TTO, positive control) in short-range bioassays (observed at 30 min). Bars topped with the same letter are not significantly different (Tukey HSD mean separation, *p* < 0.05).

In addition to the assays, to assess other mastic gum oil applications, the present study evaluated MGEOs 1–3 for their potential as attractive kairomones for male *C. capitata*. However, our laboratory bioassays indicated that the attraction of all MGEOs was significantly lower than the attraction to the positive control, tea tree essential oil (*F* = 149.0; df = 3,16; *p* < 0.001) (Figure 3).

Jang et al. [57] investigated the volatile emissions (presumedly pheromone components) from calling male *C. capitata* and quantified olfactory responses to each component using electroantennography (EAG). Myrcene was one of those components, but it elicited a lower EAG response in males as compared to females. In a study of nectarine volatiles, Light et al. [58] measured olfactory responses of *C. capitata* to myrcene and α-pinene, and both elicited weak EAG responses in males. In comparative field trials, captures of male *C. capitata* were very low with myrcene, α-pinene and β-pinene as compared to captures with the parapheromone trimedlure [59]. Recently, Niogret et al. [60] demonstrated a positive correlation between the quantity of myrcene in an essential oil and the capture of sterile male *C. capitata* in field cage bioassays. Plant phytochemicals such as α- and β-pinene are very common monoterpenoids in natural products and enantiomers of α- and β-isomers might have different biological activity. In MGEOs 1–3, there was a greater concentration of (+)-α-pinene, which might not be attractive to male *C. capitata*. We should remember that EOs are complex mixtures of terpenoids and minor constituents or enantiomer interactions between terpenoids may alter their attractancy. Individual enantiomers of α- and β-pinene need to be evaluated further to determine bioactivity.

## 3. Materials and Methods

### 3.1. Plant Material and Essential Oil Isolation

**Figure 4:**
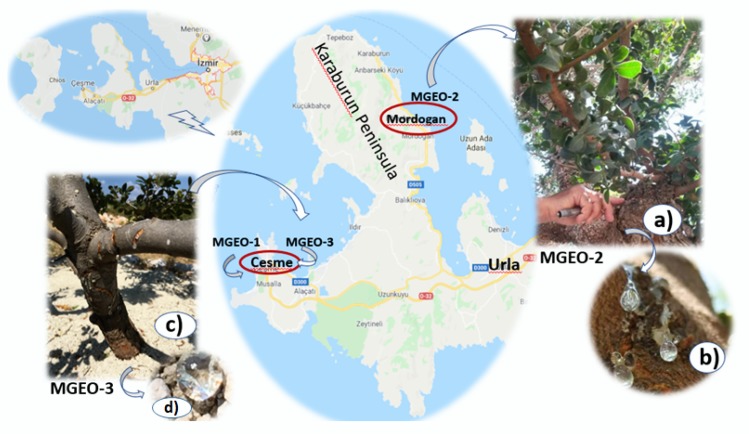
Map (www.google.com/maps) of Izmir showing regions where mastic gum samples were collected from Cesme (MGEO-1, wild and MGEO-3, cultivated) and Mordogan (MGEO-2, wild); (**a**) the tree trunk in Mordogan is “injured” with incisions; and (**b**) mastic tears flowing down from a wounded tree. Photos courtesy of A.N. (**c**) Injured bark on a cultivated tree in Cesme, Germiyan village; and (**d**) fresh mastic tear fallen to the ground. Photos courtesy of Ali Efe Karci.

At the beginning of September 2016, two mastic gum samples were collected from their natural populations in Cesme–Uzunkoy village (MGEO-1) and Mordogan (MGEO-2) (Figure 4a,b), Izmir, Turkey. The third sample, MGEO-3 was harvested from a cultivated mastic tree in Cesme, Germiyan village, Nezih Ozture Farm (Izmir) (Figure 4c,d). The cultivated mastic tree was propagated by leafy hardwood cuttings from the wild trees. Voucher samples were deposited at the Faculty of Pharmacy Herbarium, Anadolu University, Eskisehir, Turkey (Archive No: 39, 40 and 41, respectively). The harvested mastic gums were air-dried at room temperature and used for distillation in two months after harvested. Before the distillation, the mastic gums were coarsely powdered and immediately subjected to 3 h of hydrodistillation using a Clevenger-type apparatus [61]. The resulting essential oils were dried over anhydrous sodium sulfate and stored at 4 °C, until used in the bioassays and analyses.

### 3.2. Gas Chromatography (GC) and Gas Chromatography-Mass Spectrometry (GC-MS)

The gas chromatography-flame ionization detector (GC-FID) analysis was carried out using an Agilent 6890N GC system (SEM Ltd., Istanbul, Turkey). The FID temperature was 300 °C. To obtain the same elution order with GC-MS, simultaneous auto-injection was done on a duplicate of the same column applying the same operational conditions. Relative percentage amounts of the separated compounds were calculated from FID chromatograms. The analysis results are given in Table 1.

The gas chromatography-mass spectrometry (GC-MS) analysis was carried out with an Agilent 5975 GC-MSD system (SEM Ltd., Istanbul, Turkey). An Innowax FSC column (60 m × 0.25 mm, 0.25 μm film thickness) was used with helium as carrier gas (0.8 mL/min). GC oven temperature was kept at 60 °C for 10 min and programmed to 220 °C at a rate of 4 °C/min, and kept constant at 220 °C for 10 min and then programmed to 240 °C at a rate of 1 °C/min. Split ratio was adjusted at 40:1. The injector temperature was set at 250 °C. The mass spectra were recorded at 70 eV. The mass range was from *m/z* 35 to 450.

Identification of the essential oil components was carried out by comparing their relative retention times (RRT) with those of authentic samples or by comparing their relative retention index (RRI) to *n*-alkanes series. Computer matching against commercial (Wiley GC/MS Library, MassFinder 3 Library) [62,63] and in-house “Baser Library of Essential Oil Constituents” built up by genuine compounds and components of known oils, as well as MS literature data [64,65], was used for the identification.

### 3.3. Chiral Analysis of α-Pinene

Chiral analyses for α-pinene was carried out with a Rt-βDEXse 30 m, 0.32 mm × 0.25 μm column (Restek Corporation, Bellefonte, PA, USA) using a Trace GC Ultra (Thermo Scientific, Waltham, MA, USA). Helium was used as a carrier gas at 1.2 mL/min. The samples were analyzed with a split ratio of 10:1. The injector and FID temperatures were 225 and 230 °C, respectively. The temperature program was kept at 30 °C for 5 min, and then programmed to a rate of 3 °C/min to 120 °C. The standards (−)-α-pinene (Cas # 7785-26-4), and (+)-α-pinene (Cas # 7785-70-8) were acquired from Sigma-Aldrich, St. Louis, MO, USA.

### 3.4. Cell Lines and Maintenance

PANC-1 (human pancreatic carcinoma); MCF-7 (human estrogen-dependent breast adenocarcinoma); MDA-MB-231 (human estrogen-independent breast adenocarcinoma); PC-3 (human prostate adenocarcinoma); CaCo-2 (human colon colorectal adenocarcinoma); HeLa (human cervix adenocarcinoma); 253J-BV (human bladder cancer cells); A549 (human alveolar adenocarcinoma); RAW (murine macrophage cells); SK-MEL-30 (human melanoma cells); U87MG (human glioblastoma-astrocytoma); HepG2 (human liver hepatocellular carcinoma) and, as a normal cell line, HEK293 (human embryonic kidney cells) were used for testing cytotoxicity. All cell lines were purchased from *American* Type Culture Collection (ATCC, Manassas, VA, USA), only 253J-BV cells obtained from Creative Bioarray (Shirley, NY, USA). The cell lines were maintained in Dulbecco’s modified Eagle’s medium F12 (DMEM/F12), supplemented with 10% fetal bovine serum (FBS), 2 mM glutamine, 100 U/mL of penicillin and 100 µg/mL of streptomycin (Lonza, Visp, Switzerland). The cells were incubated at 37 °C in a humidified atmosphere of 5% CO_2_.The cells were sub-cultured twice a week and cells in the exponential growth phase were used in the experiments.

### 3.5. Cytotoxicity Assay

The cytotoxicity of samples was determined using a modified MTT assay [66], which detects the activity of mitochondrial reductase of viable cells. The assay principle is based on the cleavage of MTT that forms formazan crystals by cellular succinate dehydrogenases in viable cells. Insoluble formazan crystals were dissolved by the addition of dimethyl sulfoxide (DMSO) to the wells. In order to perform the cytotoxicity assay, all cell lines were cultivated for 24 h in 96-well microplates (Corning, NY, USA) with an initial concentration of 1 × 10^5^ cells/well in a humidified atmosphere with 5% CO_2_, at 37 °C. Then, the cultured cells were treated with different dilutions of samples (0.5, 5, 50 µg/mL) followed by incubation for 48 h at 37 °C. Doxorubicin (Sigma-Aldrich, St. Louis, MO, USA) was used as positive control. The optical density of the dissolved material was measured at λ = 570 nm (reference filter, λ = 620 nm) with a UV-visible spectrophotometer (Thermo Multiskan Spectrum, Waltham, MA, USA). The cell viability (%V) was determined by the following formula:

%V = [(absorbance of treated cells) − (absorbance _blank_)]/[(absorbance _control_) − (absorbance _blank_)] × 100

Cytotoxicity was expressed as a mean percentage increase relative to the unexposed control ± standard deviation (SD). The control value was set to 0% cytotoxicity. Cytotoxicity data (where appropriate) were fitted to a sigmoidal curve and a four parameters logistic model used to calculate the IC_50_, which is the concentration causing 50% inhibition in comparison to untreated controls. The mean IC_50_ is the concentration of agent that reduces cell growth by 50% under the experimental conditions and is the average from at least three independent measurements that will be reproducible and statistically significant. The IC_50_ values were reported at ±95% confidence intervals (±95% CI). This analysis was performed with Graph Pad Prism 5 (San Diego, CA, USA).

### 3.6. Morphological Studies

The morphological studies of the cells were performed with an inverted microscope (Zeiss, Jena, Germany) compared to the control group 48 h after treatment.

### 3.7. Inhibition of iNOS Activity

RAW cells were cultured in RPMI 1640 medium with 10% FBS in a humidified atmosphere with 5% CO_2_, at 37 °C. Cells were seeded in 96-well plates (1 × 10^5^ cells/well) and incubated for 24 h. After inducing with lipopolysaccharide (LPS) (1 μg/mL), dilutions of samples were added and incubated for another 24 h. Analysis was carried out as triplicates. The level of nitrite in the medium was measured by using Griess reagent (Sigma-Aldrich, St. Louis, MO, USA) in supernatants. The absorbance was measured at 540 nm. The percent inhibition of nitrite production by sample was calculated in comparison to vehicle control, and IC_50_ values were obtained from dose curves. Doxorubicin was used as a positive control.

### 3.8. In Vitro Antimicrobial Evaluation

#### 3.8.1. Microbial Strains and Culture Media

The test microorganisms were stored at −85 °C, and later at −20 °C, which were inoculated for refreshing prior the experiments by using Mueller Hinton Agar (MHA, Fluka, Germany) and Mueller Hinton Broth (MHB) (Merck, Darmstadt, Germany) and incubated at 37 °C for further 24 h. *Candida albicans* was inoculated on Potatoes Dextrose Agar (PDA) and RPMI, after sufficient growth, all microorganisms were adjusted using a densitometer (Biosan, ATC Antes, Eskişehir, Turkey) to McFarland No: 0.5 and modified according to previous guidelines [67,68,69].

#### 3.8.2. Microdilution Assay

A modified broth microdilution assay [67,69] was used to justify the activity range of the test samples. Essential oil and standard antimicrobial samples were dissolved in Dimethyl Sulfoxide (DMSO) for the initial stock solution. Essential oil dilution series were prepared in 96-well microtiter plates. Each bacterial suspension containing 10^7^ CFU/mL of the bacterial cells (100 μL) were then added to each well. Chloramphenicol and amoxicillin were from Sigma-Aldrich, St. Louis, MO, USA and used as antibacterial positive controls along with and the well containing only medium. *Candida albicans* was also prepared according to CLSI (Clinical and Laboratory Standards Institute) methods [66], where amphotericin B (Sigma-Aldrich, St. Louis, MO, USA) was used as a standard antifungal agent. The last row containing medium with microorganism was used as a negative control. After incubation at 37 °C for 24 h, the staining of viable microorganisms was performed by adding 20 μL 0.01% resazurin solution into the plates. The minimum inhibitory concentration (MIC, mg/mL) was determined as the lowest sample concentration, in which no microbial growth is visible that prevented a change in color. All experiments were repeated in triplicate and average MICs were reported comparatively using standard antimicrobial agents (MIC range: 0.125–64 μg/mL) as shown in Table 7.

### 3.9. Short-Range Attraction Bioassays with Ceratitis Capitata

Sterile male *C. capitata* were obtained from the Programa Moscamed mass rearing facility (El Pino, Guatemala), where they were irradiated as pupae 2 days prior to emergence with 95 Gy of gamma radiation from a Co^60^ source. Irradiated pupae were shipped initially to the United States Department of Agriculture-Animal and Plant Health Inspection Service (USDA-APHIS) Medfly Project (Sarasota, FL, USA), and then to the United States Department of Agriculture-Agricultural Research Service, Subtropical Horticulture Research Station (USDA-ARS SHRS) in Miami, FL, USA. All flies used for bioassays were 5 to 10 days old, sexually mature virgin males. The rearing methods were similar to those described earlier [70,71].

Laboratory bioassays were conducted to assess the potential short-range attraction of male *C. capitata* to MGEOs 1–3, using a modified version of methods reported previously [71]. All observations were carried out at room temperature in small collapsible cages (20.3 × 20.3 × 20.3 cm) into which 50 flies were introduced 1 h prior to the start of each experiment. Assays were initiated by introducing a Petri dish (53 mm diameter × 12 mm height) containing the test chemical (10 μL of a 10% dilution in acetone) applied to a filter paper disk (Whatman #1, 3.5 cm diameter). Each test used four separate cages to observe fly response to each of the three MGEOs and to a known attractant, tea tree essential oil (Essential Oil India—SAT Group, Kannauj, India) [72]. After 30 min, the response was recorded as the number of flies within a Petri dish, which was then converted to percentage of flies attracted. Tests were replicated five times, and the position of the cages was randomized between replicate runs. Flies and Petri dishes were used only once, and cages were pressure washed with hot water between tests to eliminate potential chemical residues. Analysis of variance (ANOVA), followed by mean separation with Tukey test (*p* < 0.05), was used to analyse results (Systat Software [73].

## 4. Conclusions

In this present study, detailed chemical characterization and comprehensive biological evaluation with more than ten assays were performed with MGEOs obtained from wild and cultivated mastic trees from the Izmir region, Turkey. The chemical composition of MGEOs 1 and 2 from approximately 100-year-old wild trees was very similar, while MGEO-3 from a 7-year-old cultivated tree demonstrated differences characterized with a relatively low amount of myrcene. Cultivated mastic tree gum depends on horticultural and environmental conditions; however, harvesting time, tree age, height, and the diameter of the trunk should be optimized. MGEOs 1 and 2 showed selective cytotoxicity against A559 (lung), SK-MEL-30 (melanoma), PANC-1 (pancreatic) and U87MG (glioblastoma-astrocytoma) human cells lines, whereas HeLa (cervix adenocarcinoma) cells exhibited more sensitivity to MGEOs-3 than MGEOs 1and 2. However, all samples exhibited *in vitro* cytotoxic activity against RAW264.7. MGEOs-1 to 3 showed relatively low or no inhibition against the tested microorganisms and relatively low attraction on male *C. capitata*.

Extending the initial findings of this study, it can be concluded that the relative quantity of myrcene and the enantiomeric ratios of (−)/(+)-α-pinene and (−)-α-pinene/myrcene can be used as a criteria for verifying the authenticity of mastic gum oil from commercial mastic oils, cosmetic and food products containing mastic oils. Our results suggest that MGEOs 1 and 2 are similar in chemical composition to the Chios mastic gum essential oil, and therefore can be considered as a high-quality resource for MGEO. Further detailed chemical and biological studies are needed to elaborate on the potential of *Pistacia lentiscus* L. var. *chia*, mastic gum, as other the parts of this valuable natural resource.

## Figures and Tables

**Table 1:** Chemical composition of mastic gum essential oils (MGEOs) obtained from wild and cultivated mastic trees (*Pistacia lentiscus* var. *chia*).

RRI ^a^	RRI ^b^	Compound	MGEO-1 (Wild) %	MGEO-2 (Wild) %	MGEO-3 (Cultivated) %	IM *
1014	1012^c^	Tricyclene	-	-	0.6	MS
1032	1008–1039 ^c^	α-Pinene	56.2	51.9	70.8	RRI, MS
1076	1043–1086 ^c^	Camphene	0.2	0.2	2.3	RRI, MS
1118	1085–1130 ^c^	β-Pinene	2.7	3.1	5.7	RRI, MS
1132	1098–1140 ^c^	Sabinene	0.4	0.5	0.6	RRI, MS
1135	1109–1137 ^c^	Thuja-2,4(10)-diene	tr	tr	0.1	MS
1174	1140–1175 ^c^	Myrcene	20.1	18.6	2.5	RRI, MS
1203	1212 ^d^	Limonene	1.7	2.4	2.3	RRI, MS
1218	1188–1233 ^c^	β-Phellandrene	tr	tr	-	RRI, MS
1280	1246–1291 ^c^	*p*-Cymene	0.1	0.2	0.4	RRI, MS
1348	1317–1357 ^c^	6-Methyl-5-hepten-2-one	tr	tr	tr	MS
1384	1331–1384 ^c^	α-Pinene oxide	0.5	0.6	0.4	RRI, MS
1417		4,8-Dimethyl-1,3,7-nonatriene	0.3	0.3	tr	MS
1424		*o*-Methyl anisol	0.7	0.5	0.6	RRI, MS
1429	1405–1431 ^c^	Perillene	1.9	2.2	1.5	MS
1466	1438–1480 ^c^	α-Cubebene	tr	-	tr	MS
1497	1462–1522 ^c^	α-Copaene	0.3	0.1	0.3	RRI, MS
1499		α-Campholene aldehyde	0.1	tr	0.1	MS
1535	1496–1546 ^c^	β-Bourbonene	0.3	0.1	0.4	MS
1549	1518–1560 ^c^	β-Cubebene	0.1	-	0.1	MS
1553	1507–1564 ^c^	Linalool	0.4	0.3	0.4	RRI, MS
1565	1532–1570 ^c^	Linalyl acetate	0.4	0.2	0.2	RRI, MS
1586	1545–1590 ^c^	Pinocarvone	0.2	0.2	0.2	RRI, MS
1591	1549–1597 ^c^	Bornyl acetate	0.2	-	0.7	RRI, MS
1600	1565–1608 ^c^	β-Elemene	tr	-	0.1	RRI, MS
1612	1569–1632 ^c^	β-Caryophyllene	3.3	6.8	2.8	RRI, MS
1648	1597–1648 ^c^	Myrtenal	0.2	0.2	0.2	MS
1661	1624–1668 ^c^	Alloaromadendrene	0.2	-	0.1	MS
1670	1643–1671 ^c^	*trans*-Pinocarveol	0.2	0.3	0.4	RRI, MS
1683	1665–1691 ^c^	*trans*-Verbenol	0.7	0.6	0.4	MS
1687	1663 ^e^	α-Humulene	0.7	0.9	0.5	RRI, MS
1704	1689 ^c^ 1681 ^e^	γ-Muurolene	0.4	tr	0.2	MS
1706	1694 ^c^ 1688 ^e^	α-Terpineol	0.1	0.1	0.2	RRI, MS
1709	1685–1709 ^c^	α-Terpinyl acetate	0.3	0.2	0.2	RRI, MS
1725	1696–1735 ^c^	Verbenone	0.1	0.2	0.2	RRI, MS
1733	1693–1740 ^c^	Neryl acetate	0.1	0.2	0.1	RRI, MS
1740	1686–1753 ^c^	α-Muurolene	0.2	-	0.2	MS
1772		(*Z*)-Anethole	tr	0.1	tr	RRI, MS
1773	1755 ^c^ 1749 ^e^	δ-Cadinene	0.2	-	0.2	MS
1804	1743–1808 ^c^	Myrtenol	0.2	0.1	0.2	MS
1845	1802–1846 ^c^	(*E*)-Anethole	0.3	0.2	tr	RRI, MS
1849		Calamenene	0.1	-	tr	MS
1849	1766–1849 ^c^	Cuparene	0.1	0.1	-	MS
1864	1813–1865 ^c^	*p*-Cymen-8-ol	0.1	0.1	0.2	RRI, MS
1900	1854–1928 ^c^	*epi*-Cubebol	0.1	-	tr	MS
1912		*p*-Cymen-9-ol	0.1	0.1	0.1	MS
1957	1884–1964 ^c^	Cubebo	0.2	-	0.1	MS
2001		Isocaryophyllene oxide	0.4	0.8	0.1	MS
2008	1936–2023 ^e^	Caryophyllene oxide	2.1	4.1	0.9	RRI, MS
2029	1963–2029 ^e^	Perilla alcohol	0.1	0.1	0.1	RRI, MS
2053	1980–2050 ^e^	Anisaldehyde	-	0.1	-	RRI, MS
2071	2003–2071 ^e^	Humulene epoxide-I	0.3	0.4	0.1	MS
2109		*cis*-Methyl isoeugenol	0.1	0.1	0.2	MS
2144		Dimyrcene Ia	0.1	0.1	tr	MS
2174		Dimyrcene Ib	0.1	0.1	0.1	MS
2200		*trans*-Methyl isoeugenol	0.1	tr	0.1	MS
2219		Dimyrcene IIa	0.9	1.1	0.5	RRI, MS
2269		Dimyrcene IIb	0.3	0.4	0.2	RRI, MS
2380		8α,13-Oxy-14-en-epilabdane (=epi-Manoyl oxide)	-	-	0.2	MS
2392		Caryophylla-2(12),6-dien-5β-ol (=Caryophyllenol II)	-	0.1	-	MS
2415		3,4-Dimethoxy benzaldehyde	tr	-	tr	MS
		Monoterpene Hydrocarbons	81.4	76.9	85.3	
		Oxygenated Monoterpenes	6.2	6.0	5.8	
		Sesquiterpene Hydrocarbons	5.9	8.0	4.9	
		Oxygenated Sesquiterpenes	3.1	5.4	1.2	
		Diterpenes	1.4	1.7	1.0	
		Others	1.2	1.0	0.9	
		**Total**	99.2	99.0	99.1	

^a^ RRI: Relative retention indices calculated against *n*-alkanes; ^b^ RRI: from literature on the polar column ^c^ [30], ^d^ [31], ^e^ [32]; %: calculated from flame ionization detection (FID) data; tr: Trace (< 0.1%); * IM: Identification method based on the relative retention indices (RRI) of authentic compounds on the HP Innowax column; MS, identified on the basis of computer matching of the mass spectra with those of the Wiley and MassFinder libraries and comparison with literature data; -: not detected.

**Table 2:** Essential oils from the gum of *P. lentiscus* var. *chia* collected from different geographic locations, main components, and extraction methods based on the literature survey from 1990 to 2020 [5,33,34,35,36,37,38,39,40,41,42].

Origin	Main Components (%)	Extraction Methods	Ref.
Spain	α-pinene (78.6), β-pinene (3.3), myrcene (3.2)	Hex	[33]
Greece	α-pinene (58.9, 77.1), myrcene (0.2, 12.3), linalool (3.7, 0.5)	SD	[34]
Commercial	α-pinene (66.5), myrcene (8.3), β-pinene (3.3)	SD	[35]
Commercial	α-pinene (33.7–72.8), myrcene (3.8–63.5)	-	[36]
Chios Island	α-pinene (72.1), myrcene (16.5), β-pinene (2.9)	SDE	
Turkey (Fethiye)	β-pinene (38.7), α-pinene (21.7), pinocarvone (5.3), limonene (3.8), *n*-nonanal (3.5)	D	[37]
Commercial	α-pinene (63.3), myrcene (25), β-pinene (3.3)	-	[38]
CMGGA	α-pinene (40.9), (Z,Z)-farnesol (11.9), β-caryophyllene (5.3), myrcene (9.0), β-pinene (1.7)	EtOH soluble part	
Greece	α-pinene (25.6), verbenone (14), *p*-cymene (9.8), verbenene (8.6), 2-methylanisole (6.5), 1,2-dimethyl-4-ethyl benzene (6), pinocarvone (4.9), myrtenal (3)	SPME	[39]
CMGGA	α-pinene (59.2–87.1), myrcene (4.7–27.6), β-pinene (1.6–3.6), β-caryophyllene (0.1–4.9)	-	[40]
Greece	α-pinene (67.5), β-pinene (2.8), verbenone (2.6), *trans*-pinocarveol (2.5), *p*-mentha-1,5-dien-8-ol (2.4), myrcene (1.1)	HD	[5]
	α-pinene (34.9-46), verbenone (3.2–5.5), *trans*-verbenol (5.9–7.1), caryophyllene oxide (2.5–3.9), β-caryophyllene (1.8–3.8), *trans*-pinocarveol (1.6–2.2), β-pinene (1.4–2.1)	SFE	
Chios Island	α-pinene, β-pinene, camphene, myrcene, 2-nonanone, perillene, linalool, terpinen-4-ol	SPME-SBSE	[41]
CMGGA	α-pinene (67.7), myrcene (18.8), β-pinene (3.1)	DD	[42]

CMGGA: Chios Mastic Gum Growers Association; SDE; simultaneous distillation–extraction; HD: hydrodistillation; Hex: extracted by hexane; SD: steam distillation; SFE: supercritical fluid extraction; D: distillation; SPME: Solid-phase microextraction; SBSE: stir bar sorptive extraction; DD: dry distillation; -: Not available

**Table 3:** Ratios of (−)/(+)-α-pinene and (−)-α-pinene/myrcene for the MGEOs-1–3.

Experimental Results Mean ± SD * (*n* = 3)	(−)/(+)- α-Pinene	Myrcene/(+)- α-Pinene	(−)-α-Pinene/ Myrcene	Myrcene/ α-Pinene	β-Pinene/(+)- α-Pinene	β-Pinene/ Myrcene
MGEO-1 (wild)	0.0058 ± 0.0009	0.20 ± 0	0.029 ± 0.004	0.36	0.027 ± 0	0.029 ± 0.004
MGEO-2 (wild)	0.0051 ± 0.0005	0.19 ± 0.009	0.027 ± 0.002	0.36	0.031 ± 0	0.074 ± 0.002
MGEO-3 (cultivated)	0.0060 ± 0.0007	0.025 ± 0	0.24 ± 0.028	0.04	0.023 ± 0	0.24 ± 0.028
**Literature Values [40]**						
45 Authentic Chios MGEOs (min-max)	0.0055–0.010	0.06–0.34	0.019–0.11	0.06–0.34	0.020–0.038	
Average Chios MGEOs	0.0071 ± 0014	0.18 ± 0.06	0.045 ± 0018		0.027 ± 0.0062	
19 Commercial products	0–0.67		0–0.74			

* SD = Standard deviation.

**Table 4:** IC_50_ values (mean ± SD, μg/mL) of MGEOs 1 to 3 for cell lines after 48 h exposure to different MGEO concentrations.

Cell Lines	IC_50_ μg/mL			
	MGEO-1 (Wild)	MGEO-2 (Wild)	MGEO-3 (Cultivated)	Doxorubicin *
PANC-1	14.76 ± 2.68	18.05 ± 3.72	46.87 ± 3.70	26.48 ± 2.16
MCF-7	47.45 ± 1.45	39.52 ± 6.85	38.69 ± 4.33	20.25 ± 0.41
MDA-MB-231	23.08 ± 0.55	26.55 ± 0.16	12.40 ± 0.39	19.33 ± 0.50
PC3	15.3 ± 0.29	30.67 ± 5.72	19.54 ± 0.09	6.35 ± 0.38
CaCo-2	31.74 ± 5.84	49.91 ± 0.40	47.60 ± 3.72	10.25 ± 0.26
HeLa	20.11 ± 4.15	18.81 ± 0.73	7.621 ± 1.91	2.14 ± 0.26
253J-BV	15.96 ± 1.08	10.82 ± 1.25	12.14 ± 1.93	2.50 ± 1.30
A549	7.02 ± 1.94	5.77 ± 1.65	19.93 ± 1.23	5.57 ± 1.09
RAW264.7	7.62 ± 0.51	9.20 ± 2.64	5.84 ± 0.68	1.23 ± 0.53
SK-MEL-30	5.25 ± 0.33	5.25 ± 0.81	13.31 ± 2.53	2.09 ± 0.18
U87MG	11.71 ± 2.74	6.12 ± 1.49	23.42 ± 3.21	6.38 ± 1.05
HepG2	25.19 ± 2.51	44.83 ± 1.42	47.47 ± 2.54	5.63 ± 0.07
HEK293	15.74 ± 0.08	17.83 ± 3.00	17.66 ± 1.58	1.19 ± 0.17

* Positive control.

**Table 5:** Percentage of nitric oxide (NO) inhibition (mean ± SD) observed with MGEOs 1–3 in lipopolysaccharide (LPS)-activated RAW264.7 cells incubated for 24 h with different MGEO concentrations.

Concentration (μg/mL)	MGEO-1 (Wild)	MGEO-2 (Wild)	MGEO-3 (Cultivated)	Doxorubicin *
50	105.83 ± 3.15	106.79 ± 0.89	105.19 ± 1.35	
5	11.35 ± 0.89	17.39 ± 1.35	6.89 ± 0.89	
0.5	15.48 ± 11.25	7.53 ± 3.59	10.07 ± 6.29	
20				94.06 ± 2.65
2				6.47 ± 1.23
0.2				4.55 ± 0.89

* Positive control.

**Table 6:** Inducible nitric oxide synthase (iNOS) IC_50_ values (mean ± SD, μg/mL) of MGEOs 1 to 3 in LPS-activated RAW264.7 cells incubated for 24 h with different concentrations of EO.

Samples	IC_50_ (μg/mL)
MGEO-1 (wild)	11.6 ± 0.9
MGEO-2 (wild)	13.75 ± 0.4
MGEO-3 (cultivated)	13.85 ± 0.5
Doxorubicin *	7.2 ± 0.1

* Positive control.

**Table 7:** Antimicrobial activity of MGEOs 1-3 (MIC *, mg/mL).

Samples	*E. coli* NRRL B-3008	*S. aureus* ATCC 6538	*S. typhimurium* ATCC 13311	*L. monocytogenes* ATCC 19111	*K. pneumoniae* NCTC 9633	*C. albicans* ATCC 90028
MGEO-1 (wild)	>10	5	2.5	5	5	1.25
MGEO-2 (wild)	>10	>10	>10	>10	5	2.5
MGEO-3 (cultivated)	>10	>10	5	>10	>10	2.5
Chloramphenicol **	8	8	8	4	8	-
Amoxicillin **	0.25	>32	<0.062	<0.062	<0.062	
Amphotericin B **	-	-	-	-	-	0.125

* MIC: Minimum inhibitory concentration, ** positive controls’ MICs are expressed in µg/mL, -: not tested.

## Supplementary Materials

The following are available online, **Figure S1.** Viability of cancer and healthy cell lines following sample treatment for 48 h. Cell viability was determined by MTT assay, control was exposed to vehicle only which was taken as 100% viability. Data are expressed as mean ± SD. MGEO-1 (A), MGEO-2 (B), MGEO-3 (C), and positive control Doxorubicin (D) Doxorubicin. PANC-1, human pancreatic carcinoma cells; MCF-7, human estrogen-dependent breast adenocarcinoma cells; MDA-MB-231, human estrogen-independent breast adenocarcinoma cells; PC3, human prostate epithelial cells; CaCo-2, human colon carcinoma epithelial cells; HEK293, human embryonic epithelial kidney cell; HeLa, human cervical epithelial carcinoma cells; 253J-BV, human bladder cancer cells, A549, human lung epithelial cells; RAW 264.7, murine macrophage cells; SK-MEL-30, human melanoma cells; U87MG, human glioblastoma-astrocytoma epithelial-like cells; HepG2, human liver hepatocellular carcinoma cells. **Figure S2.** (A) HEK293, untreated, (B) HEK293, 50 µg/mL MGEO-1, (C) HEK293, 50 µg/mL MGEO-2, (D) HEK293, 50 µg/mL MGEO-3, (F) CaCo-2, untreated, (G) CaCo-2, 50 µg/mL MGEO-1, (H) CaCo-2, 50 µg/mL MGEO-2, (I) CaCo-2, 50 µg/mL MGEO-3, (J) HeLa, untreated, (K) HeLa, 50 µg/mL MGEO-1, (L) HeLa, 50 µg/mL MGEO-2, (M) HeLa, 50 µg/mL MGEO-3. **Figure S3.** (A) 253J-BV, untreated, (B) 253J-BV, 50 µg/mL MGEO-1, (C) 253J-BV, 50 µg/mL MGEO-2, (D) 253J-BV, 50 µg/mL MGEO-3, (E) MCF-7, untreated, (F) MCF-7, 50 µg/mL MGEO-1, (G) MCF-7, 50 µg/mL MGEO-2, (H) MCF-7, 50 µg/mL MGEO-3, (I) MDA-MB-231, untreated, (J) MDA-MB-231, 50 µg/mL MGEO-1, (K) MDA-MB-231, 50 µg/mL MGEO-2, (L) MDA-MB-231, 50 µg/mL MGEO-3. **Figure S4.** (A) PANC-1, untreated, (B) PANC-1, 50 µg/mL MGEO-1, (C) PANC-1, 50 µg/mL MGEO-2, (D) PANC1, 50 µg/mL MGEO-3, (E) PC3, untreated, (F) PC3, 50 µg/mL MGEO-1, (G) PC3, 50 µg/mL MGEO-2, (H) PC3, 50µg/mL MGEO-3, (I) A549, untreated, (J) A549, 50 µg/mL MGEO-1, (K) A549, 50 µg/mL MGEO-2, (L) A549, 50 µg/mL MGEO-3. **Figure S5.** (A) RAW 264.7, untreated, (B) RAW 264.7, 50 µg/mL MGEO-1, (C) RAW 264.7, 50 µg/mL MGEO-2, (D) RAW 264.7, 50 µg/mL MGEO-1, (E) SK-MEL-30 untreated, (F) SK-MEL-30, 50 µg/mL MGEO-1, (G) SK-MEL-30, 50 µg/mL MGEO-2, (H) SK-MEL-30, 50 µg/mL MGEO-3, (I) U87MG, untreated, (J) U87MG, 50 µg/mL MGEO-1, (K) U87MG, 50 µg/mL MGEO-2, (L) U87MG, 50 µg/mL MGEO-3. **Figure S6.** (A) HepG2, untreated, (B) HepG2, 50 µg/mL MGEO-1, (C) HepG2, 50 µg/mL MGEO-2, (D) HepG2, 50 µg/mL MGEO-3.
