# Supplementary material for: Chemical Characterization and Biological Activity of the Mastic Gum Essential Oils of Pistacia lentiscus var. chia from Turkey
Source: Molecules. 2020 May 2;25(9):2136. doi: 10.3390/molecules25092136 (PMC7248992; doi:10.3390/molecules25092136)
Supplement: Supplementary file 1 [file molecules-25-02136-s001.pdf]

molecules

MDPI

Supplementary Materials

Chemical Characterization and Biological Activity of the Mastic Gum Essential Oils of *Pistacia lentiscus* var. *chia* from Turkey†

Nurhayat Tabanca[1*], Ayse Nalbantsoy[2*], Paul E. Kendra[1], Fatih Demirci[3,4], Betul Demirci[3]

1 United States Department of Agriculture, Agricultural Research Service, Subtropical Horticulture Research Station (SHRS), Miami, FL 33158, USA; Paul.Kendra@usda.gov
2 Department of Bioengineering, Faculty of Engineering, Ege University, Bornova, Izmir 35100, Turkey
3 Department of Pharmacognosy, Faculty of Pharmacy, Anadolu University, Eskisehir 26470, Turkey; demircif@gmail.com (F.D.); betuldemirci@gmail.com (B.D.)
4 Faculty of Pharmacy, Eastern Mediterranean University, Famagusta 99628 N. Cyprus

* Correspondence: Nurhayat.Tabanca@usda.gov (N.T.); analbantsoy@gmail.com (A.N.).; Tel.: +1-(786)-5737077 (N.T.); +90-(232)-3115807 (A.N.)
† This work was produced by US government employees and is in the public domain in the US.

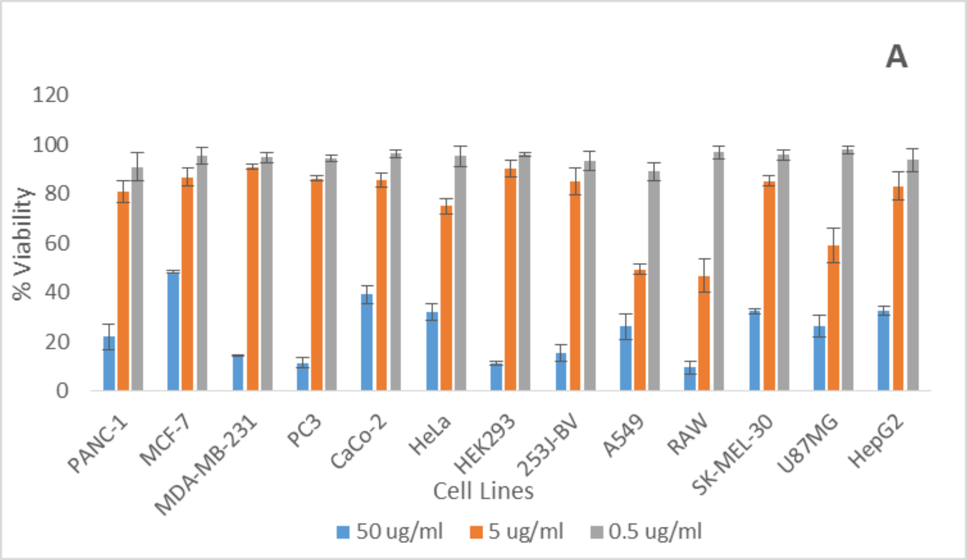

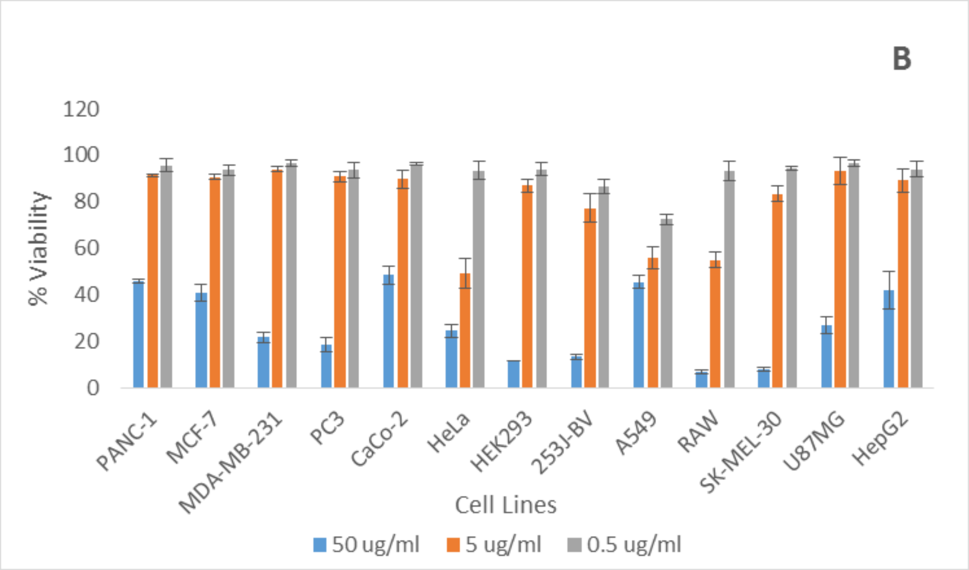
B
% Viability
120
100
80
60
40
20
0
PANC-1
MCF-7
MDA-MB-231
PC3
CaCo-2
HeLa
HEK293
253J-BV
A549
RAW
SK-MEL-30
U87MG
HepG2
Cell Lines
50 ug/ml
5 ug/ml
0.5 ug/ml

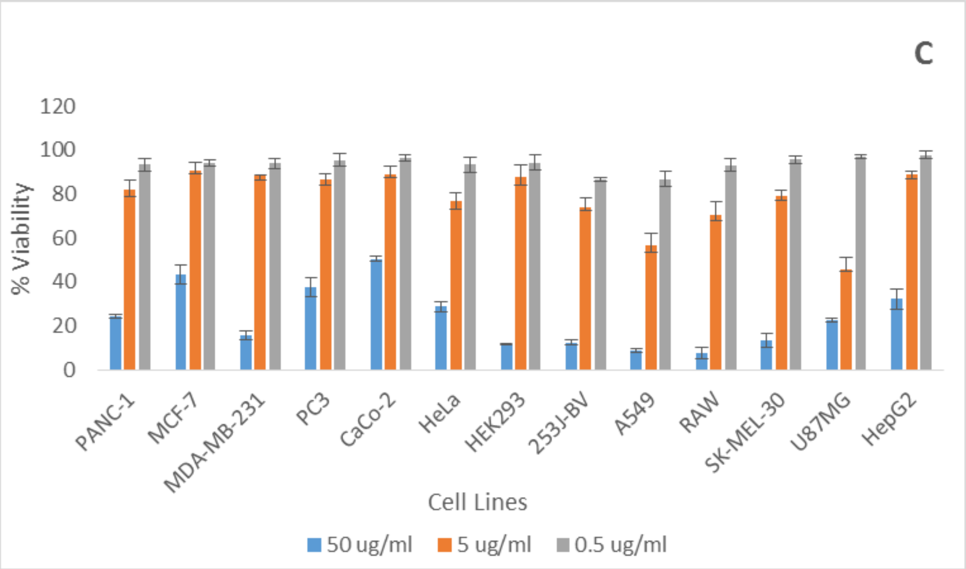
C
% Viability
120
100
80
60
40
20
0
PANC-1
MCF-7
MDA-MB-231
PC3
CaCo-2
HeLa
HEK293
253J-BV
A549
RAW
SK-MEL-30
U87MG
HepG2
Cell Lines
50 ug/ml
5 ug/ml
0.5 ug/ml

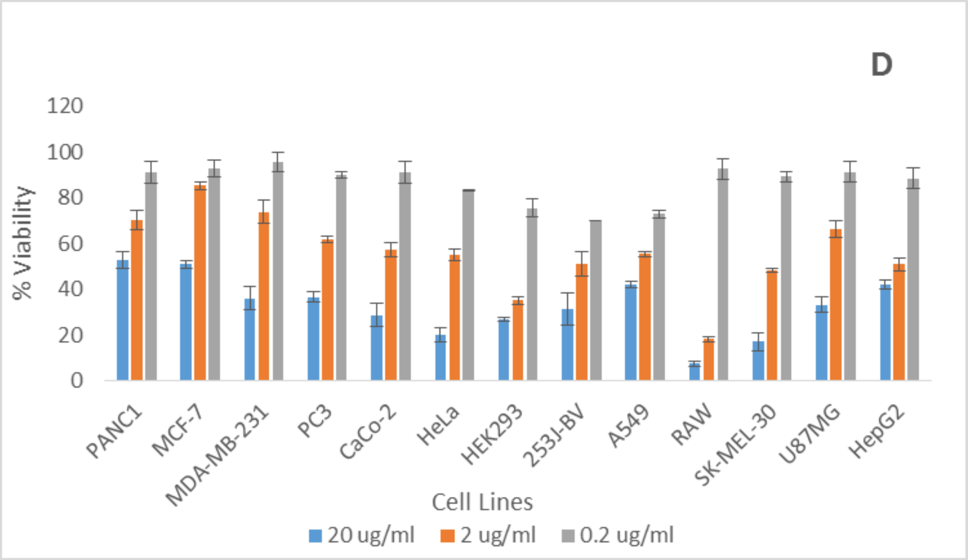

Figure S1. Viability of cancer and healthy cell lines following sample treatment for 48 h. Cell viability was determined by MTT assay, control was exposed to vehicle only which was taken as 100% viability. Data are expressed as mean ± SD. MGEO-1 (**A**), MGEO-2 (**B**), MGEO-3 (**C**), and positive control Doxorubicin (**D**) Doxorubicin. PANC-1, human pancreatic carcinoma cells; MCF-7, human estrogen-dependent breast adenocarcinoma cells; MDA-MB-231, human estrogen-independent breast adenocarcinoma cells; PC3, human prostate epithelial cells; CaCo-2, human colon carcinoma epithelial cells; HEK293, human embryonic epithelial kidney cell; HeLa, human cervical epithelial carcinoma cells; 253J-BV, human bladder cancer cells, A549, human lung epithelial cells; RAW 264.7, murine macrophage cells; SK-MEL-30, human melanoma cells; U87MG, human glioblastoma-astrocytoma epithelial-like cells; HepG2, human liver hepatocellular carcinoma cells.

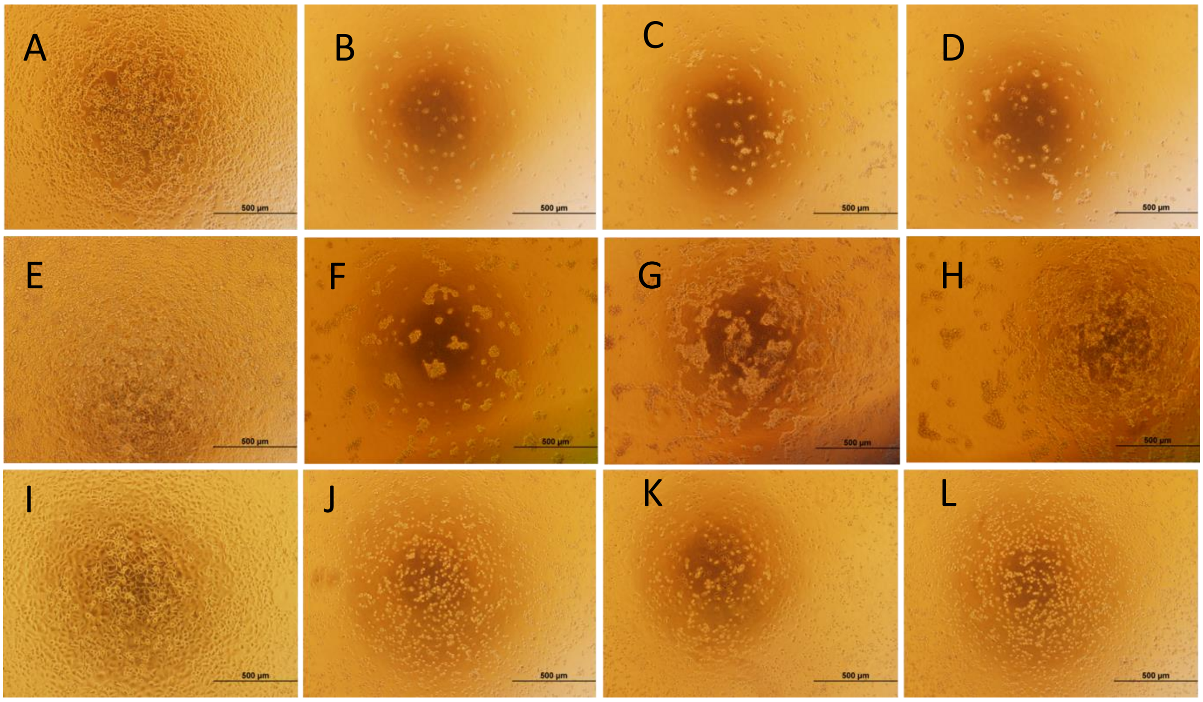

Figure S2. (A) HEK293, untreated, (B) HEK293, 50 µg/mL MGEO-1, (C) HEK293, 50 µg/mL MGEO-2, (D) HEK293, 50 µg/mL MGEO-3, (F) CaCo-2, untreated, (G) CaCo-2, 50 µg/mL MGEO-1, (H) CaCo-2, 50 µg/mL MGEO-2, (I) CaCo-2, 50 µg/mL MGEO-3, (J) HeLa, untreated, (K) HeLa, 50 µg/mL MGEO-1, (L) HeLa, 50 µg/mL MGEO-2, (M) HeLa, 50 µg/mL MGEO-3.

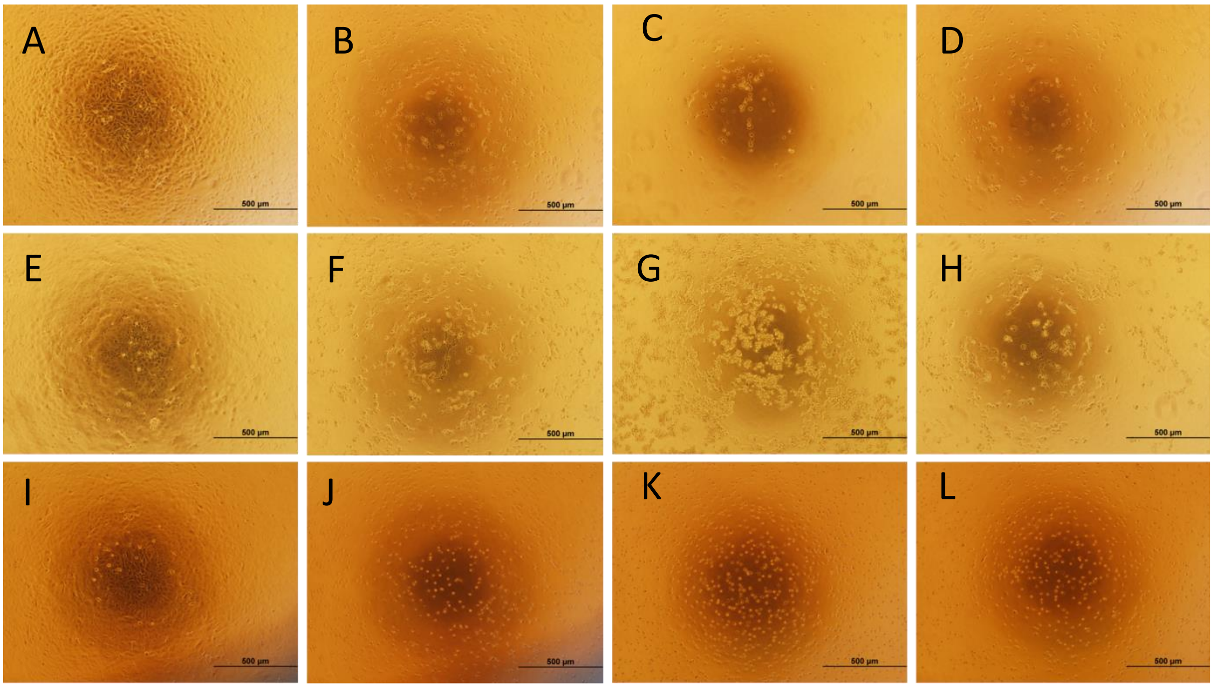

Figure S3. (A) 253J-BV, untreated, (B) 253J-BV, 50 µg/mL MGEO-1, (C) 253J-BV, 50 µg/mL MGEO-2, (D) 253J-BV, 50 µg/mL MGEO-3, (E) MCF-7, untreated, (F) MCF-7, 50 µg/mL MGEO-1, (G) MCF-7, 50 µg/mL MGEO-2, (H) MCF-7, 50 µg/mL MGEO-3, (I) MDA-MB-231, untreated, (J) MDA-MB-231, 50 µg/mL MGEO-1, (K) MDA-MB-231, 50 µg/mL MGEO-2, (L) MDA-MB-231, 50 µg/mL MGEO-3.

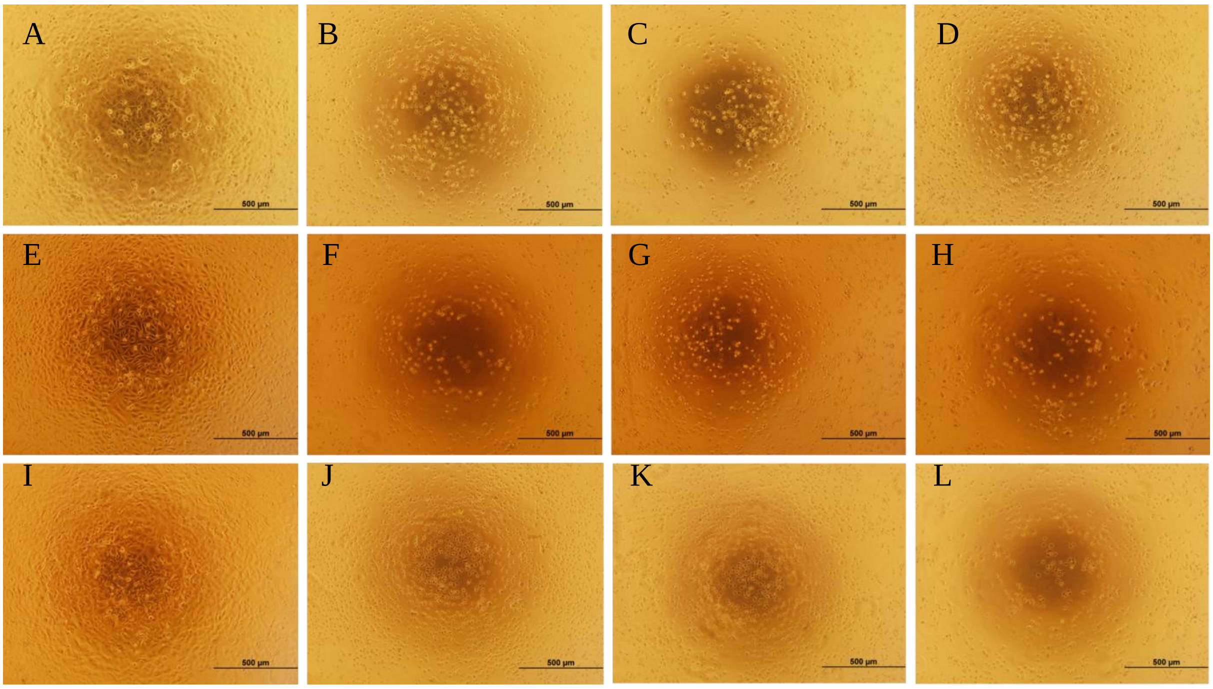

Figure S4. (A) PANC-1, untreated, (B) PANC-1, 50 µg/mL MGEO-1, (C) PANC-1, 50 µg/mL MGEO-2, (D) PANC-1, 50 µg/mL MGEO-3, (E) PC3, untreated, (F) PC3, 50 µg/mL MGEO-1, (G) PC3, 50 µg/mL MGEO-2, (H) PC3, 50 µg/mL MGEO-3, (I) A549, untreated, (J) A549, 50 µg/mL MGEO-1, (K) A549, 50 µg/mL MGEO-2, (L) A549, 50 µg/mL MGEO-3.

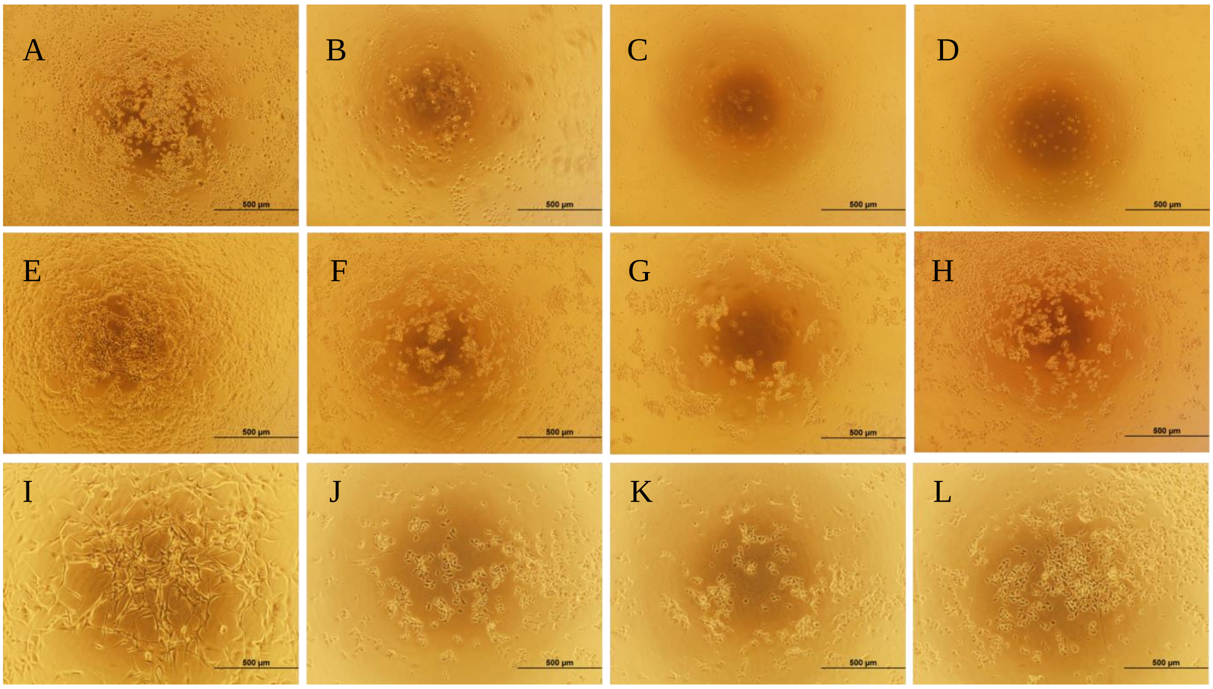

Figure S5. (A) RAW 264.7, untreated, (B) RAW 264.7, 50 μg/mL MGEO-1, (C) RAW 264.7, 50 μg/mL MGEO-2, (D) RAW 264.7, 50 μg/mL MGEO-1, (E) SK-MEL-30 untreated, (F) SK-MEL-30, 50 μg/mL MGEO-1, (G) SK-MEL-30, 50 μg/mL MGEO-2, (H) SK-MEL-30, 50 μg/mL MGEO-3, (I) U87MG, untreated, (J) U87MG, 50 μg/mL MGEO-1, (K) U87MG, 50 μg/mL MGEO-2, (L) U87MG, 50 μg/mL MGEO-3.

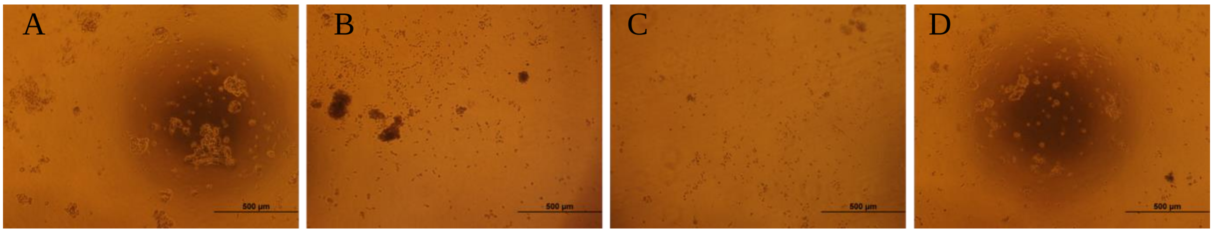

Figure S6. (A) HepG2, untreated, (B) HepG2, 50 μg/mL MGEO-1, (C) HepG2, 50 μg/mL MGEO-2, (D) HepG2, 50 μg/mL MGEO-3.
